# The Influence of 5-HTTLPR Genotype on the Association between the Plasma Concentration and Therapeutic Effect of Paroxetine in Patients with Major Depressive Disorder

**DOI:** 10.1371/journal.pone.0098099

**Published:** 2014-05-23

**Authors:** Tetsu Tomita, Norio Yasui-Furukori, Taku Nakagami, Shoko Tsuchimine, Masamichi Ishioka, Ayako Kaneda, Norio Sugawara, Sunao Kaneko

**Affiliations:** Department of Neuropsychiatry, Graduate School of Medicine, Hirosaki University, Hirosaki, Japan; Penn State College of Medicine, United States of America

## Abstract

**Introduction:**

The efficacy of treatment with selective serotonin reuptake inhibitors in patients with major depressive disorder (MDD) can differ depending on the patient's serotonin transporter-linked polymorphic region (5-HTTLPR) genotype, and the effects of varying plasma concentrations of drugs can also vary. We investigated the association between the paroxetine plasma concentration and clinical response in patients with different 5-HTTLPR genotypes.

**Methods:**

Fifty-one patients were enrolled in this study. The Montgomery-Asberg Depression Rating Scale (MADRS) was used to evaluate patients at 0, 1, 2, 4, and 6 weeks. The patients' paroxetine plasma concentrations at week 6 were measured using high-performance liquid chromatography. Additionally, their 5-HTTLPR polymorphisms (alleles S and L) were analyzed using a polymerase chain reaction with specific primers. We divided the participants into two groups based on their L haplotype: the SS group and the SL and LL group. We performed single and multiple regression analyses to investigate the associations between MADRS improvement and paroxetine plasma concentrations or other covariates for each group.

**Results:**

There were no significant differences between the two groups with regard to demographic or clinical data. In the SS group, the paroxetine plasma concentration was significantly negatively correlated with improvement in MADRS at week 6. In the SL and LL group, the paroxetine plasma concentration was significantly positively correlated with improvement in MADRS at week 6 according to the results of the single regression analysis; however, it was not significantly correlated with improvement in MADRS at week 6 according to the results of the multiple regression analysis.

**Conclusion:**

Among patients with MDD who do not respond to paroxetine, a lower plasma concentration or a lower oral dose of paroxetine might be more effective in those with the SS genotype, and a higher plasma concentration might be more effective in those with the SL or LL genotype.

## Introduction

Despite continued efforts to optimize the pharmacological treatment of individuals with major depressive disorder (MDD), the efficacy and tolerability of medications remain highly variable. Many previous reports have revealed that clinical heterogeneity [1], [2], diagnostic uncertainty [3], and environmental [4], social and genetic factors [5], [6] play important roles in determining interindividual differences in the therapeutic and toxic effects of antidepressants.

Numerous researchers have attempted to establish a clear relationship between the plasma concentrations of psychotropic drugs and patients' clinical response to these drugs [7]–[17]. Therapeutic ranges have been established for several major psychotropic drug classes, including mood stabilizers (e.g., lithium) [7], [9], tricyclic antidepressants (TCAs) [10], [12], [15], and ‘atypical’ antipsychotics (e.g., clozapine) [17]. The American Psychiatric Association Task Force on the Use of Laboratory Tests in Psychiatry (1985) concluded that, when treating patients with MDD, there was robust evidence for the utility of plasma concentration measurements of imipramine, desmethylimipramine (desipramine), and nortriptyline, but not of other TCAs [11]. On the basis of these findings, therapeutic drug monitoring has been shown to be clinically useful for a number of tricyclic antidepressants [18], [19]. In the case of selective serotonin reuptake inhibitors (SSRIs), therapeutic concentration ranges have been demonstrated [20], but because these drugs have a wide therapeutic index, toxicity is not typically a major concern.

Paroxetine is an SSRI that is widely used to treat mental disorders, including MDD, panic disorders, and obsessive-compulsive disorder [21], [22]. Large interindividual variations have been observed in the pharmacokinetics of paroxetine in adults [23]–[25]. Although many previous studies have failed to identify an association between the plasma concentration of paroxetine and its therapeutic efficacy [26]–[28], some studies have indicated a possible association [29], and monitoring paroxetine concentrations has provided some benefits [30]. Gex-Fabry et al. (2007) reported that higher paroxetine concentrations might result in an acute improvement in depressive symptoms [31], and Gilles et al. (2005) suggested a threshold paroxetine serum concentration (39.1 ng/ml), above which unfavorable effects on MDD symptoms were observed [32]. We previously reported that the plasma concentration of paroxetine was negatively associated with patient improvement and that clinical responses occurred at an upper threshold of 64.2 ng/ml [33]. Thus, findings on the relationship between the paroxetine plasma concentration and clinical efficacy remain inconsistent in patients with MDD.

Recently, many studies have investigated the associations between clinical response in MDD and polymorphisms in the serotonin transporter-linked polymorphic region (5-HTTLPR) of the serotonin transporter gene SLC6A4. Some reports have shown that the L allele or the LL genotype was associated with improved clinical response in MDD. The L allele or the LL genotype has been associated with increased expression of 5-HTT mRNA [34], [35] and greater serotonin uptake [36]. In addition to these biological roles, previous studies have reported that the L allele and the LL genotype were correlated with an enhanced response to escitalopram [37] and SSRIs [38] and that the population of MDD remitters included a larger proportion of individuals with the LL genotype [39]. In contrast, the S allele is correlated with lower expression and activity of 5-HTT [40]–[42] and might be a risk factor for developing MDD [43]. Previous studies have reported that the S allele was associated with worse response [44], [45], higher remission [46], lower tolerability, and more frequent side effects of MDD treatments [47].

Thus, the efficacy of SSRI treatment in patients with MDD can differ depending on the 5-HTTLPR genotype, and the efficacy of measuring plasma concentrations can also differ. In this study, we investigated the association between the paroxetine plasma concentration and clinical response in patients grouped according to their 5-HTTLPR polymorphisms.

## Methods

### Patients

Male and female patients aged 18 to 70 years with a diagnosis of MDD based on the Diagnostic and Statistical Manual of Mental Disorders, Fourth Edition, were eligible for this study. In addition, the patients were required to score >20 points on the Montgomery-Asberg Depression Rating Scale (MADRS) [48]. The MADRS consists of 10 items, each of which is scored on a scale that ranges from 0 to 6. The patients were required to be free of medications, including any psychotropic agents, for at least 1 month prior to the start of the study. The following exclusion criteria were applied: clinically significant abnormal laboratory or electrocardiography findings; a history of a mental disorder other than MDD (e.g., bipolar affective disorder, schizophrenia, epilepsy, alcoholism, or drug abuse); and the presence of any clinically significant organic or neurological disease. This study was approved by the Ethics Committee of Hirosaki University Hospital, and the patients provided written, informed consent prior to participating.

A total of 120 patients were initially enrolled in the study, and 89 patients completed the study (34 men and 55 women). A total of 31 patients withdrew from the study for the following reasons: 17 experienced severe side effects; four exhibited undetectable plasma drug concentrations; seven did not complete the required hospital visits for unknown reasons; and three withdrew consent for personal reasons. Of the patients who completed the study, 51 consented to DNA analysis and were included in the data analysis. The patients' age, expressed as the mean ± standard deviation (SD), was 46.3±13.8 years, and the mean body weight was 55.3±9.6 kg.

### Protocol

During the first visit (in the morning), blood samples (10 ml) were obtained after a 30-min rest. Two well-trained psychiatrists performed pretreatment clinical status assessments using the structured interview guide for the MADRS (SIGMA) [49] to assess depressive symptoms and the Udvalg for Kliniske Undersogelser (UKU) side effect rating scale [50].

A dose of 20 mg/day of paroxetine (Paxil, GlaxoSmithKline, Tokyo, Japan) was administered at 8 PM for the first week, and the dose was increased to 40 mg/day during the 2nd to the 6th weeks. If mild side effects (a UKU score of 1) were observed, the dose was maintained. The paroxetine dose was reduced if moderate side effects were observed (a UKU score of 2), and its administration was discontinued if severe side effects (a UKU score of 3) occurred. No other drugs were administered, except for diazepam (2–5 mg/day, n = 19) for anxiety, brotizolam (0.25 mg/day, n = 20 or 4 mg/day, n = 17) for insomnia, and sennoside (12–48 mg/day, n = 12) as a laxative to treat constipation. Blood samples were obtained during treatment weeks 1, 2, and 6. Clinical symptoms were evaluated using the MADRS and the UKU side effect rating scale during treatment weeks 1, 2, 4, and 6.

### Assays for paroxetine

The patients' paroxetine plasma concentrations at week 6 were measured using a high-performance liquid chromatography (HPLC) method developed in our laboratory [51], [52]. In brief, the extraction was performed as follows: 500 µl of 0.5 M NaOH, 100 ml of an internal standard solution (trifluperidol, 200 mg/ml), and 100 ml of methanol were added to a 2000 ml plasma sample. The tubes were then mixed by vortexing for 10 s, and 5 ml of n-heptane-chloroform (70∶30 v/v) was added as an extraction solvent. After 10 min of shaking, the mixture was centrifuged at 2500 *g* for 10 min at 4°C, and the organic phase was evaporated to dryness in a vacuum at 40°C (TAITEC VC-960, Shimadzu, Kyoto, Japan). The residue was dissolved in 500 ml of the mobile phase, and 400 ml was injected into the HPLC system. The HPLC system consisted of LC-10AT high-pressure pumps, a CTO-10AVP column oven, a Workstation CLASS-VP chromatography integrator, an SPD-10AVP, an SIL-10ADVP (with a 500-ml injection volume), and a column (STR-ODS II C18 1504.6, 3 mm), all of which were from Shimadzu (Shimadzu, Kyoto, Japan). The mobile phase consisted of phosphate buffer (0.02 M, pH 4.6), acetonitrile, and 60% perchloric acid (57.25∶42.5∶0.25, v/v/v). The lower limits of detection and quantification were 0.5 and 1.0 ng/ml, respectively, and the values of the intraassay and interassay coefficients of variation were <10% at all calibration curve concentrations (1.0–150 ng/ml) of paroxetine.

### Genotyping

The 5-HTTLPR polymorphism (alleles S and L) was analyzed using the polymerase chain reaction (PCR) with the primers GGCGTTGCCGCTCTGAATGC (forward) and GAGGGACTGAGCTGGACAACCAC (reverse), which resulted in products with lengths of 484 bp (S) and 528 bp (L). The PCR reaction mixture contained a total volume of 15 µl, consisting of 50–100 ng of genomic DNA; 10× PCR buffer 1; 1.5 mM MgCl_2_; 0.2 mM each of dATP, dCTP, and dTTP; 0.15 mM dGTP; 0.05 mM 7-deaza-dGTP; 10 pM of each primer; and 1 unit of Taq polymerase (Roche, Tokyo, Japan).

The cycling conditions were as follows: an initial denaturation at 94°C for 2 min; 30 cycles of denaturation at 95°C for 30 s, annealing at 63°C for 30 s, and extension at 72°C for 1 min; and a final extension at 72°C for 10 min. The iCycler (Bio-Rad Laboratories, Inc., Hercules, CA, USA) was used. The PCR products were separated using an automated capillary electrophoresis genetic analyzer (HDA-GT12, eGene, Inc., CA, USA).

### Data analyses and statistics

There were 36 SS participants, 14 SL participants, and one LL participant. The Hardy-Weinberg equilibrium for the 5-HTTLPR polymorphism was tested using the chi-square test. The data did not deviate significantly from Hardy-Weinberg equilibrium (χ^2^ = 0.07, p = 0.79). We divided the participants into two groups based on their L haplotype: the SS group and the SL and LL group.

We defined responders as patients with improvements of >50% in the MADRS score after 6 weeks of treatment (over the baseline score). We defined remitters as patients with MADRS scores <10 at the 6-week treatment time point.

The patient demographics and clinical data were compared using t-tests and chi-square tests or Fisher's exact test. We attempted to perform an analysis of covariance (ANCOVA) to test for effects of plasma concentration and 5-HTTLPR genotype on MADRS improvement at week 6. After the ANCOVA, we performed a single regression analysis to investigate the association between MADRS improvement at week 6 and the paroxetine plasma concentration. Additionally, we performed multiple regression analyses to investigate the associations between MADRS improvements at week 6 and the paroxetine plasma concentration or other covariates (sex, age, height, and body weight) for each group.

A p-value <0.05 was considered statistically significant. All of the analyses were performed using SPSS Statistics, version 21 (SPSS Japan Inc., Tokyo, Japan).

## Results

### Participant characteristics

**Figure 1:**
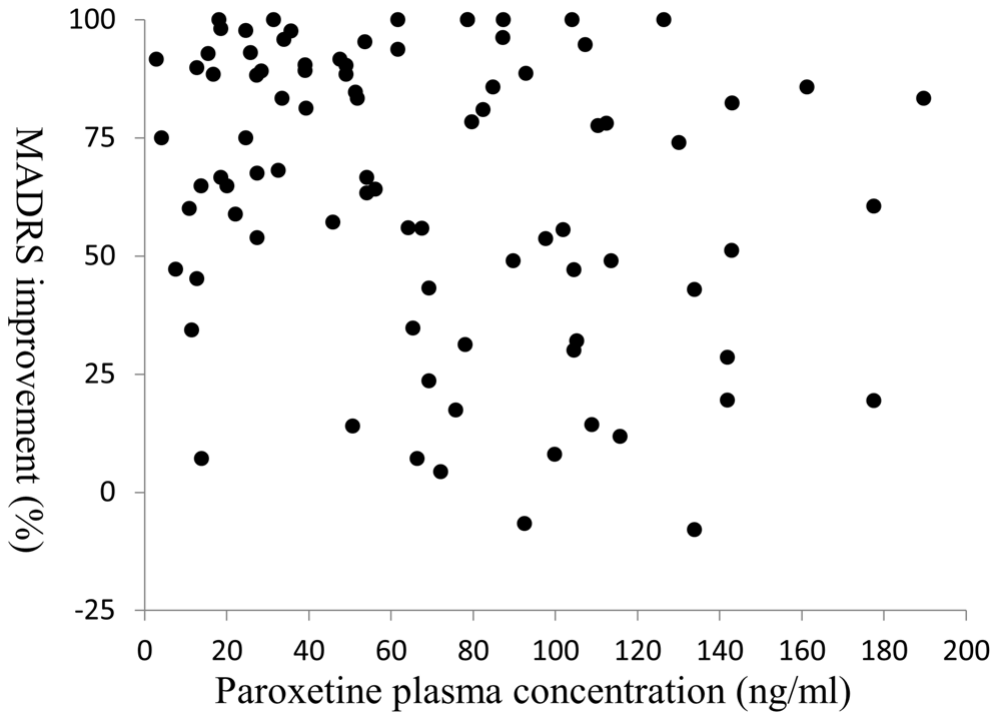
The relationship between paroxetine plasma concentration and the MADRS improvement rate: The data from each subject are plotted.

The demographic and clinical characteristics of the participants are shown in Table 1. The relationship between the plasma concentration of paroxetine and the MADRS improvement rate is shown in Figure 1.

**Table 1:** Subject demographics.

		SS (n = 36)	SL and LL (n = 15)	p-value
Sex (male: female)^a^		13∶23	3∶12	0.333
Age		47.0±14.4	44.5±12.8	0.572
Height (cm)		161.2±9.7	158.3±4.7	0.181
Body weight (kg)		56.1±10.5	53.4±7.5	0.398
Baseline MADRS score		39.2±9.5	39.8±7.8	0.817
MADRS improvement (%)	1W	16.4±26.3	13.4±19.5	0.698
	2W	42.4±28.2	35.4±27.8	0.426
	4W	55.0±29.8	44.9±35.1	0.300
	6W	60.7±33.3	53.2±41.1	0.494
Responders^b^		n = 25 (69.4%)	n = 9 (60.0%)	0.514
Remitters^b^		n = 16 (44.4%)	n = 7 (46.7%)	0.884
Plasma concentration of paroxetine (ng/ml)		66.4±47.3	74.4±46.5	0.581

a : Fisher's exact test;

b : Chi-squared test.

The numbers of responders and remitters were 26 and 16 in the SS group and nine and seven in the SL and LL group, respectively. There were no significant differences between the groups regarding any of the patient characteristics, including sex, age, height, body weight, baseline MADRS score, MADRS improvement at each week (over the baseline score), numbers of responders and remitters, or paroxetine plasma concentrations.

### The influence of 5-HTTLPR genotype on the association between improvement in MADRS score and paroxetine plasma concentration

For the ANCOVA, we assessed the homogeneity of the regression slope assumption using the MADRS improvement rate as the dependent variable, the plasma concentration of paroxetine as the covariate, and 5-HTTLPR genotype (SS group or SL and LL group) as the fixed factor. The results revealed a significant interaction between the 5-HTTLPR genotype and the fixed factors (df = 2, F = 99.407, p = 0.002); therefore, we could not perform ANCOVA. In the next analysis, we analyzed the participants divided in two groups according to their 5-HTTLPR genotype.

**Figure 2:**
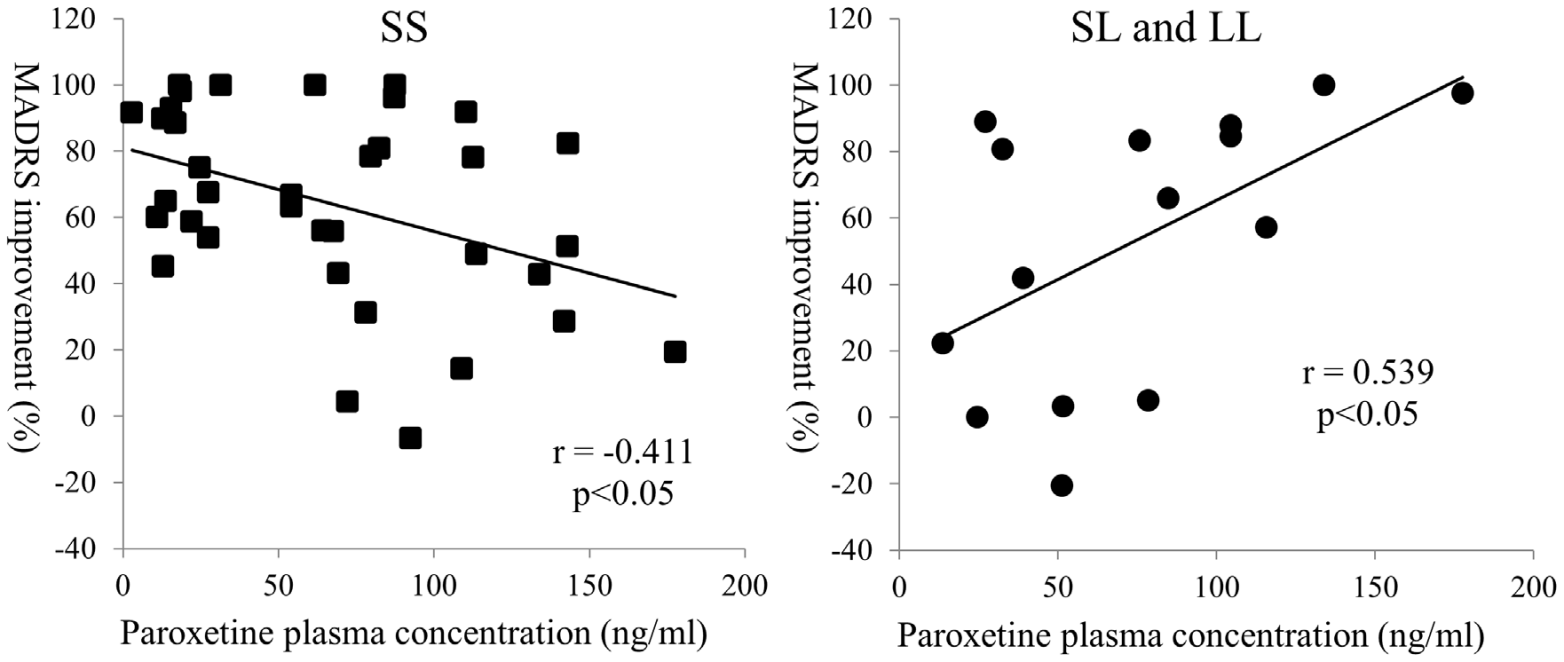
The results of single regression analysis in MADRS improvement and the paroxetine plasma concentration.

Figure 2 shows the results of the single regression analysis of the improvements in MADRS in both groups.

In the SS group, the paroxetine plasma concentration was significantly negatively correlated with improvement in MADRS at week 6. In contrast, in the SL and LL group, the paroxetine plasma concentration was significantly positively correlated with improvement in MADRS at week 6.

### The influence of 5-HTTLPR genotype on the associations between improvement in MADRS and covariates

Table 2 shows the results of multiple regression analyses of improvements in MADRS in both groups. Sex, height, body weight, and paroxetine plasma concentration were not significantly associated with improvement in MADRS at week 6 in either group.

**Table 2:** Multiple regression analysis of the improvement in MADRS at week 6.

	SS			SL and LL		
	coefficient	t-value	p-value	coefficient	t-value	p-value
Sex	−0.006	−0.022	0.983	−0.236	−0.644	0.540
Age	0.142	0.853	0.401	0.222	0.715	0.498
Height (cm)	−0.333	−1.050	0.303	0.475	1.617	0.150
Body weight (kg)	−0.025	−0.095	0.925	−0.477	−1.278	0.242
Plasma concentration of PXT (ng/ml)	−0.405	−2.512	0.019*	−0.211	−0.717	0.496

PXT: paroxetine, *: p<0.05.

In the SS group, only the paroxetine plasma concentration showed a significant association with improvement in MADRS at week 6. However, in the SL and LL group, the paroxetine plasma concentration was not significantly associated with improvement in MADRS at week 6.

## Discussion

In this study, we compared patients grouped based on their 5-HTTLPR polymorphisms in terms of the association between the paroxetine plasma concentration and clinical response. The results of single and multiple regression analyses indicated that the plasma concentration of paroxetine was negatively correlated with improvement in MADRS at week 6 in MDD patients with the SS 5-HTTLPR genotype; however, the single regression analysis results indicated that those factors were positively correlated in MDD patients with the SL and LL 5-HTTLPR genotypes. This is the first report to demonstrate significant negative and positive associations between the paroxetine plasma concentration and treatment efficacy in MDD patients divided according to 5-HTTLPR genotype. Based on our results, a lower plasma concentration or a lower oral dose of paroxetine might be more effective in patients with the SS genotype, and a higher plasma concentration might more effective in patients with the SL or LL genotype. There was a difference between the results of the single and multiple regression analyses. Some independent variables in the multiple regression analysis may have contradicted the significance of the association between the improvement in MADRS and the plasma concentration of paroxetine in patients with the SL or LL genotype.

The results of Dreimüller et al. showed that among MDD patients with an L allele, those with higher SSRI plasma concentrations exhibited better responses to treatment than those with lower concentrations [53]. In our study, the results of the multiple regression analysis indicated that there was no association between treatment outcome and paroxetine plasma concentration in the carriers of the L allele. The small number of subjects with the LL genotype might have affected this result. However, our results provide support for the effectiveness of measuring paroxetine plasma concentrations in patients with specific 5-HTTLPR genotypes.

It is unclear why the paroxetine plasma concentration was negatively correlated with improvement in MADRS in the SS group. David et al. reported that the decrease in 5-HTT function associated with the S allele might result in a lifelong increase in 5-HT and the desensitization and downregulation of 5-HT1A receptors [54]. Gilles et al. suggested that the plasma concentration of paroxetine in responders was significantly lower than in non-responders [32]. The pharmacological characteristics of paroxetine and this desensitization of 5-HTT receptors by the S allele of 5-HTTLPR might underlie the negative correlation between the paroxetine plasma concentration and improvement in MADRS in the SS group.

Many previous studies have reported an increased response to SSRI treatment in patients carrying the L allele compared with the S allele; however, our results did not reveal a significant superiority of the SL and LL genotypes with regard to improvement in MADRS, and the mean weekly improvement in MADRS in the SL and LL group was smaller than in the SS group. The majority of the previous studies of 5-HTTLPR have investigated Caucasians and have reported improved responses to SSRI treatment for MDD in patients carrying the L 5-HTTLPR allele and worse responses in patients carrying the S 5-HTTLPR allele. However, several studies of 5-HTTLPR in Asian populations have reported that S allele carriers with MDD exhibited a better response to SSRI treatment compared with L allele carriers [55], [56]. Umene-Nakano et al. studied Japanese patients treated with sertraline, and Won et al. studied Korean patients treated with escitalopram. It is possible that the function or clinical role of the 5-HTTLPR polymorphism in patients with MDD may differ on the basis of race.

In this study, there were some limitations. First, the frequencies of the S and L alleles (84.3% and 15.7%, respectively) in this study were slightly different from those in previous studies, and the sample size was small. Kunugi et al. and Umene-Nakano et al. reported S and L allele frequencies of approximately 79% and 21%, respectively [55], [57]. The number of LL subjects in the present study was small. Additional, important information might be discovered in larger samples of subjects with the LL genotype. Second, we investigated only the L and S alleles of 5-HTTLPR. Other studies have examined the 5-HTT single nucleotide polymorphisms (SNPs) rs25531 and STin2 in addition to 5-HTTLPR [58]–[61]. If we had investigated these SNPs, our study might have yielded more important results. Third, this study was preliminary. We showed a correlation between clinical improvement and the paroxetine plasma concentration in patients with MDD, but our results lack clinical indices and do not indicate the therapeutic reference range for paroxetine plasma concentrations. Further studies are required to investigate details of the correlations between clinical improvement and the plasma concentration of paroxetine in patients with MDD.

In conclusion, we found negative and positive correlations between the plasma concentrations of paroxetine and improvement in MADRS at 6 weeks in patients with MDD who were divided based on 5-HTTLPR genotype. For patients with MDD who do not respond to paroxetine, a lower plasma concentration or a lower oral dose of paroxetine might be more effective for those with the SS genotype, and a higher plasma concentration might be more effective in those with the SL or LL genotype. Further investigations are required before the results from this study can be applied in the clinic.
